# Protection or constraint? A phenomenological study of the learning experience among doctors receiving postgraduate medical education during the COVID-19 pandemic

**DOI:** 10.1186/s12909-026-08782-y

**Published:** 2026-02-12

**Authors:** I-Ting Hwang, Wei-Hung Lin, Po-Wei Chiu, Po-Ching Huang, Ying-Nien Chiu, Servet Üztemur, Ming-Ta Hsieh, Ru-Yi Huang, Jung-Sheng Chen, Marc N. Potenza, Mark D. Griffiths, Chung-Ying Lin

**Affiliations:** 1 Department of Occupational Therapy, College of Medicine, National Cheng Kung University, 1 University Rd, Tainan, 701401 Taiwan; 2 Department of Internal Medicine, College of Medicine, National Cheng Kung University Hospital, National Cheng Kung University, 1 University Rd, Tainan, 701401 Taiwan; 3 Department of Emergency Medicine, College of Medicine, National Cheng Kung University Hospital, National Cheng Kung University, 1 University Rd, Tainan, 701401 Taiwan; 4 Department of Physiotherapy, School of Nursing and Health Sciences, Hong Kong Metropolitan University, Hong Kong SAR, Hong Kong, China; 5 Institute of Allied Health Sciences, College of Medicine, National Cheng Kung University, 1 University Rd, Tainan, 701401 Taiwan; 6 Department of Turkish and Social Sciences Education, Faculty of Education, Anadolu University, Yunus Emre Kampüsü 26470 Tepebaşı, Eskişehir, Türkiye; 7 Department of Family Medicine and Community Medicine, E-Da Hospital, I-Shou University, No. 1, Yida Rd., Kaohsiung, 824005 Taiwan; 8 School of Medicine, College of Medicine, I-Shou University, No. 8, Yida Rd., Kaohsiung, 824005 Taiwan; 9 Division of Family Medicine, School of Medicine, Taipei Tzu Chi Hospital, Buddhist Tzu Chi Medical Foundation, Tzu Chi University, Hualien, Taiwan; 10 Data Science Degree Program, National Taiwan University and Academia Sinica, Taipei, Taiwan; 11 Department of Medical Research, E-Da Hospital, I-Shou University, Kaohsiung, 824005 Taiwan; 12 Department of Psychiatry, Yale School of Medicine, 333 Cedar St, New Haven, CT 06510 USA; 13 Connecticut Mental Health Center, 34 Park St, New Haven, CT 06519 USA; 14 Connecticut Council on Problem Gambling, 75 Charter Oak Avenue, Suite 1-309, Hartford, CT 06106 USA; 15 Child Study Center, Yale School of Medicine, 333 Cedar St, New Haven, CT 06510 USA; 16 Department of Neuroscience, Yale University, New Haven, CT 06520 USA; 17 Wu Tsai Institute, Yale University, New Haven, CT 06520 USA; 18 Psychology Department, Nottingham Trent University, 50 Shakespeare St, Nottingham, NG1 4FQ UK; 19 Biostatistics Consulting Center, National Cheng Kung University Hospital, College of Medicine, National Cheng Kung University, 1 University Rd, Tainan, 701401 Taiwan

**Keywords:** Postgraduate training, Learning experiences, Medical education, COVID-19 pandemic, Phenomenological research

## Abstract

**Background:**

The COVID-19 pandemic considerably impacted medical education, and adopting new education approaches was needed to address public health emergencies. Doctors undergoing postgraduate medical education (PGY doctors) were likely to encounter complex challenges stemming from the pandemic. However, there is limited understanding of how their learning was affected from their perspective. Therefore, the present study explored the learning experiences of PGY doctors during the pandemic.

**Methods:**

In the present qualitative study, 24 PGY doctors participated in five focus groups, which took place during the pandemic in Taiwan (August to November 2022). Participants were invited to share their perceptions of encountering the COVID-19 pandemic during their training and how the pandemic affected their learning experiences. The audio recordings of the focus groups were transcribed verbatim and analyzed using the Framework Method.

**Results:**

The data suggested that the COVID-19 pandemic had four key impacts on the learning context (i.e., the hospital environment in Taiwan): (a) facing the uncertainty of COVID-19 infection risk, (b) new regulations imposed by pandemic-related prevention policies, (c) lack of essential equipment and personnel in the hospital, and (d) reduced exposure to other diseases observed and treated during the pandemic. Two main learning challenges were identified. First, PGY doctors faced unexpected changes in the breadth and depth of learning during the pandemic. Second, PGY doctors experienced heightened psychological stress in their learning due to the pandemic.

**Conclusions:**

The results suggest that the COVID-19 pandemic brought new challenges for PGY doctors’ learning experience. The breadth and depth of their learning unavoidably changed during the pandemic, and they experienced a mismatch between expected and actual capabilities. To some extent, the *protections* they were given as medical students became *constraints* when they transitioned into the role of a PGY doctor. It is important for medical educators to consider how to create and provide alternative forms of modeling to enhance potential missed learning experiences and increase the capability and confidence of PGY doctors.

**Clinical trial registration:**

Not applicable.

**Supplementary Information:**

The online version contains supplementary material available at 10.1186/s12909-026-08782-y.

## Background

The coronavirus disease 2019 (COVID-19) pandemic posed considerable challenges to medical education globally [1, 2]. Prior systematic reviews indicated that medical education faced several COVID-19-related challenges, including the widespread cancellation of clinical training, reduced availability of clinical mentors, and increased psychological stress across all levels of medical trainees [3–5]. These multi-faceted challenges may shape trainees’ learning experiences and carry potential long-term implications for their future clinical practice [6, 7].

However, the impact of the pandemic was not the same across all levels of medical training. For medical students, the adoption of new teaching approaches (e.g., remote learning) may have effectively mitigated some disruptions in their education [8–10]. In contrast, newly licensed doctors who have just completed their undergraduate education and entered postgraduate medical education (i.e., doctors undergoing postgraduate medical education [PGY doctors]) may face a more complex situation. For PGY doctors, not having sufficient opportunities for “hands-on” experience in a supervised environment may have led to significant gaps in their competence and confidence [5, 11, 12]. Therefore, it is important for medical educators to develop a deep understanding of the specific types of challenges PGY doctors encountered during the COVID-19 pandemic.

Despite the large body of medical education literature that emerged during the pandemic, most studies have focused on educational activities that can be readily adapted to virtual formats, such as undergraduate teaching and continuing medical education [13–15]. Even within studies emphasizing hands-on experience, the focus has largely been on how virtual simulations can enhance surgical skills, with comparatively less attention given to other critical clinical skills [16, 17]. However, given that the core learning objective of PGY training is to develop overall clinical competence (including patient care and management, communication and interpersonal skills, and professionalism), such training is difficult to replace with virtual formats. Moreover, existing research on postgraduate training during the COVID-19 pandemic has largely focused on specialty-specific residents who already had some level of clinical stability [3, 17–20], with relatively little attention given to newly licensed, undifferentiated PGY doctors. While quantitative surveys have identified common stressors among PGY doctors [3, 21], there is limited qualitative research that provides an in-depth understanding of how the pandemic influenced the learning process from PGY doctors’ perspectives.

In Taiwan, the impact of the COVID-19 pandemic on medical education occurred under specific conditions. The Taiwanese government adopted relatively proactive prevention measures, such as implementing border controls and community surveillance systems, to reduce the potential spread of COVID-19 [22, 23]. In 2020, while other countries experienced multiple outbreaks, Taiwan remained in a stable situation with no domestic cases for approximately 8 months [24] and was regarded by the international community as a country with a relatively successful experience in epidemic prevention [25]. During the pandemic, the Ministry of Health and Welfare provided guidance that enabled PGY medical education to continue in modified forms [26]. Because of these measures, PGY medical education in Taiwan was able to maintain a level of operation that was unique globally. These conditions offered a valuable opportunity to explore how PGY doctors adapted to pandemic-related changes when the system was strained but still functioning.

To better understand the potential impacts of the COVID-19 pandemic on PGY medical education, there is a clear need for an in-depth exploration based on the lived experiences of those at the frontline. Developing this understanding is crucial for ensuring that medical educators are prepared with robust educational alternatives for PGY doctors in future public health emergencies. Therefore, the present study explored PGY doctors’ learning experiences during the COVID-19 pandemic, focusing specifically on how the pandemic impacted their learning context and their perceived learning challenges.

## Methods

### Research design

The present study explored PGY doctors’ learning experiences and perspectives regarding the COVID-19 pandemic in Taiwan, and was designed according to the phenomenology design using a qualitative research methodology. Phenomenology aims to gain a rich understanding of the meanings that individuals attribute to a concept or phenomenon [27]. The focus of phenomenological studies is how individuals perceive phenomena, how they describe them, how they feel about them, how they make sense of them, how they judge and remember them, and how they talk about them with others [28]. Phenomenological research focuses entirely on personal perceptions and narratives and seeks to explore experiences from the perspective of the “insider” [29]. The phenomena addressed in the present study were PGY doctors’ perceptions, experiences, and perspectives regarding the impact of the COVID-19 pandemic on their educational learning.

### Participants

**Fig. 1:**
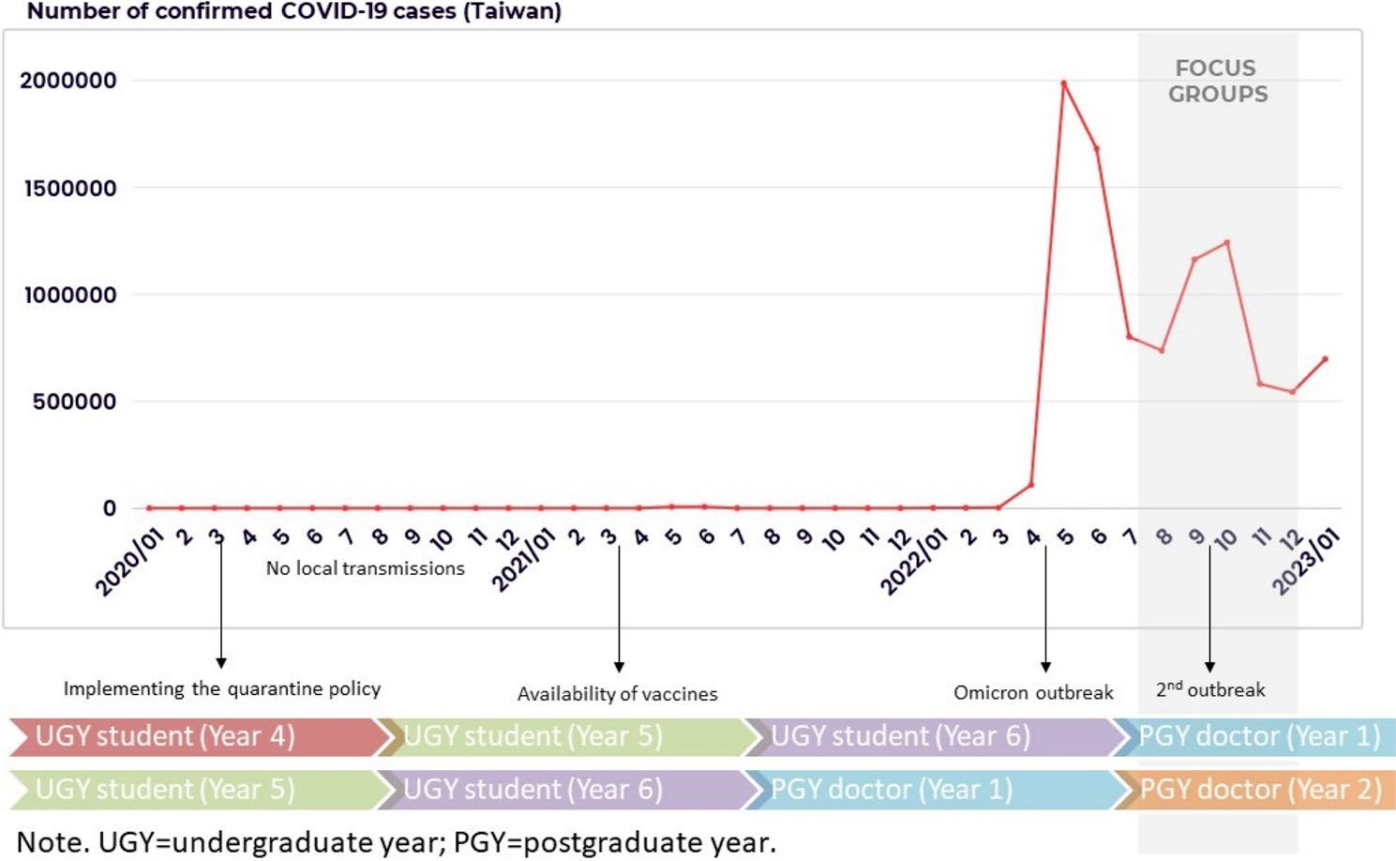
The timeframe of participants’ training stages and the number of confirmed cases in Taiwan from January 2020 to January 2023

In Taiwan, undergraduate year (UGY) medical education spans six years. After graduation, most individuals take the National Board Exam, which grants a medical license and allows these individuals to legally practice medicine in Taiwan, and then enter a two-year postgraduate medical education program as PGY doctors. Upon completing two years of postgraduate medical education, they may apply for residency training in a specific specialty. Participants in the present study were recruited through criterion sampling, a qualitative research sampling technique [30]. The criteria for being a participant were (a) having graduated from a medical school within the past two years and (b) being currently enrolled in postgraduate medical education in Taiwan. One of the research team members (Y-NC) attended the training orientation for PGY doctors in August 2022 and invited potential individuals to participate in the study. Twenty-four individuals (17 males and 7 females) who worked in a teaching hospital in Southern Taiwan participated. Among the 24 participants, 18 were in their first year of PGY medical education (Y1), and six were in their second year (Y2) when they participated in the focus groups. The COVID-19 pandemic situation in Taiwan and the participants’ training stages are shown in Fig. 1.

### Data collection

A semi-structured interview guide was developed based on the existing literature, including review papers on the impact of the COVID-19 pandemic on medical training [3–5, 7] and one qualitative study [6], as well as consultations with experts in the medical education field in Taiwan, and a pilot group of six PGY doctors who were not included in the main study. The interview guide focused on the participants’ perceptions of encountering the COVID-19 pandemic during their training, how the pandemic affected their clinical work and learning experiences, and their perceived challenges and support during the pandemic. The detailed interview guide can be found in the Supplementary Materials.

Five focus groups were held between August and November 2022 with four to six participants in each group. At the start of each focus group, the interviewer (Y-NC) explained the study purpose and content of written consent forms to ensure that the participants were fully aware that the group would be audio-recorded, how the data would be stored, and that their decision to be part of the study was voluntary. After obtaining written informed consent from all the participants, all focus group discussions were audio-recorded. The main role of the interviewer was to encourage participants to share their thoughts, and when appropriate, the interviewer summarized participants’ contributions and invited additional input to ensure no perspectives were overlooked. The average duration of each focus group was approximately one hour. The present study protocol was reviewed and approved by the National Cheng Kung University Human Research Ethics Committee (HREC-E-111-325-2), and was conducted in accordance with the Declaration of Helsinki.

### Data analysis

The data analysis was guided by the Framework Method [31], which includes seven steps. First, *transcription* (Step 1), the focus groups were transcribed verbatim and checked by the group leader (Y-NC). Second, *familiarization with the interview* (Step 2), Y-NC read and re-read the transcripts, exploring participants’ responses to gain a comprehensive understanding of their learning experiences. Third, *coding* (Step 3), Y-NC conducted line-by-line coding and then labeled the key concepts. Four, *developing a working analytical framework* (Step 4), Y-NC and I-TH put the initial codes into a working analytical framework (i.e., codebook), revised the analytical framework based on multiple rounds of discussion, and provided definitions of each code. Fifth, *applying the analytical* framework (Step 5), Y-NC systematically applied the finalized analytical framework to all the transcripts. Following this step, each code encompassed one or more quotes from different participants. To enhance understanding of each code, two authors reviewed the full set of quotes associated with each code, and selected those that best illustrated its core meaning. The categories, codes, definitions, and example quotes are provided in Table 1. Six, *charting data into the framework matrix* (Step 6), Y-NC and I-TH created a matrix where the columns were categories and codes, and the rows were different participants. Finally, *interpreting the data with the matrix* (Step 7), guided by the study aim, the research team explored relationships between categories to identify key impacts of the COVID-19 pandemic on the learning context and generate potential themes related to participants’ perceived learning challenges. The interpretations were then presented to a domain expert familiar with medical education.

**Table 1:** The analytical framework and key findings (categories, codes, definitions, and example quotes)

Categories	Codes	Definition	Example quote
Feelings of uncertainties	Fear of infection	Concerns about getting infected by the virus and the potential impacts on others	*“When I was a [UGY] student*,* I worried that if I get infected*,* I might cause problems for my family members.”* (Y1-2)
Infection control measures	Home quarantine	Experiences related to the mandatory home quarantines	*“The regulation was that the person wouldn’t be able to attend the board exam if the person got infected and was required to follow the mandatory home quarantine for the first seven days.”* (Y1-12)
	Use of protective equipment	Experiences related to wearing protective gear, including masks, gloves, and gowns, to reduce infection risk	*“It took a lot of time putting on and taking off the isolation gown…Sometimes*,* it was also hard to do some checking procedures or identify arteries accurately with gloves on.”* (Y2-4)
	Pre-admission screening	Experiences related to implementing the COVID-19 test for all new patients	*“The procedure of the pre-admission screening was very trivial and lengthy. You needed to get an order first…after getting the sample*,* you had to send it to the lab and wait for the results…It was a long time for the patient to wait before getting the treatment.”* (Y1-17)
	COVID-19-specialized wards	Experiences related to the specialized wards that were designed to care for COVID-19 confirmed cases only	*“In the separated ward*,* you always need two people to work as a pair simultaneously. The one inside the ward first needs to call the one outside the ward*,* and the one outside can then use computers to write orders or arrange beds.”* (Y1-1)
	Ward segregation policy	Experiences related to the separation of wards and related personnel	*“We [PGY doctors] were assigned to different wards*,* and we were not allowed to move across wards.”* (Y1-10)
Limited resources during the pandemic	Necessary equipment	Experiences related to a lack of necessary equipment, including protective equipment and other hardware	*“Even basic things like testing a patient’s blood glucose could be hard to complete when I needed to do it in the COVID-19 specialized ward. I tried to bring everything I needed with me*,* but sometimes I still couldn’t find the equipment*,* such as the test strips.”* (Y1-1)
	Vaccines	Experiences related to the lack of COVID-19 vaccines	*“I wasn’t too worried about the infection risk…I think one of the reasons why was that we could get the vaccines as [PGY] doctors.”* (Y2-6)
	Human resource	Experiences related to a lack of supporting individuals, including formal and informal caregivers	*“Compared to when family members could help care for the patients*,* during the pandemic*,* it was a lot harder to monitor all the patients closely and simultaneously.”* (Y1-17)
Changes in learning opportunity	Types of diseases	The COVID-19 pandemic brought changes in the types of diseases	*“I was expecting to see more types of diseases when receiving training in the Infectious Disease Department. However*,* at that time*,* all fevers turned out to be COVID-19.”* (Y1-2)
	Clinical experience	Changes in the frequencies of clinical experiences (e.g., numbers of surgeries and practice settings)	*“During the pandemic*,* I was receiving training in the Surgical Department. Due to the outbreak*,* all the surgeries that were not urgent were postponed. The amount and types of patients I could observe were both reduced.”* (Y1-7)
	Training schedules	Changes or cancellations in the regular training schedules (e.g., differences in the duration or fields)	*“Because my training got changed to the COVID-19-specialized wards*,* it ended up that I didn’t get the opportunity to receive training in one of the medical specialties.”* (Y2-1)
	Interaction patterns with senior staff	Changes in the amount of time and interaction characteristics with senior staff	*“When I was working in the COVID-19 specialized ward*,* I needed to report to multiple attending physicians in one day because each patient’s attending physicians would be different.”* (Y2-3)
Role differences	Expectations for UGY students	Participants’ perceptions of others’ expectations toward UGY students (e.g., limited level of engagement in the clinical settings)	*“Basically*,* students wouldn’t interact with patients with high risks. During the outbreak*,* the protection regulation was quite strict. They [UGY students] were not even allowed to go to the emergency department.”* (Y1-7)
	Expectations for PGY doctors	Participants’ perceptions of others’ expectations toward PGY doctors (e.g., front-line workers who shared actual clinical responsibilities)	*“Many responsibilities are different…PGY doctors were at the front line. We had to do the observations…We had to make the judgment call when the situation changed.”* (Y2-2)
Psychological stress	Anxiety	A feeling of worry, nervousness, or unease, often in response to uncertainty	*“I was worried about taking the work shifts when I just became a [PGY] doctor…because I didn’t have any experience of taking work shifts during the [UGY] stage.”* (Y1-11)
	Self-doubt	Lack of confidence in one’s abilities, decisions, or contributions	*“I still have a big self-doubt about my ability to diagnose or make decisions…because I really wasn’t good enough.”* (Y1-1)

## Results

**Fig. 2:**
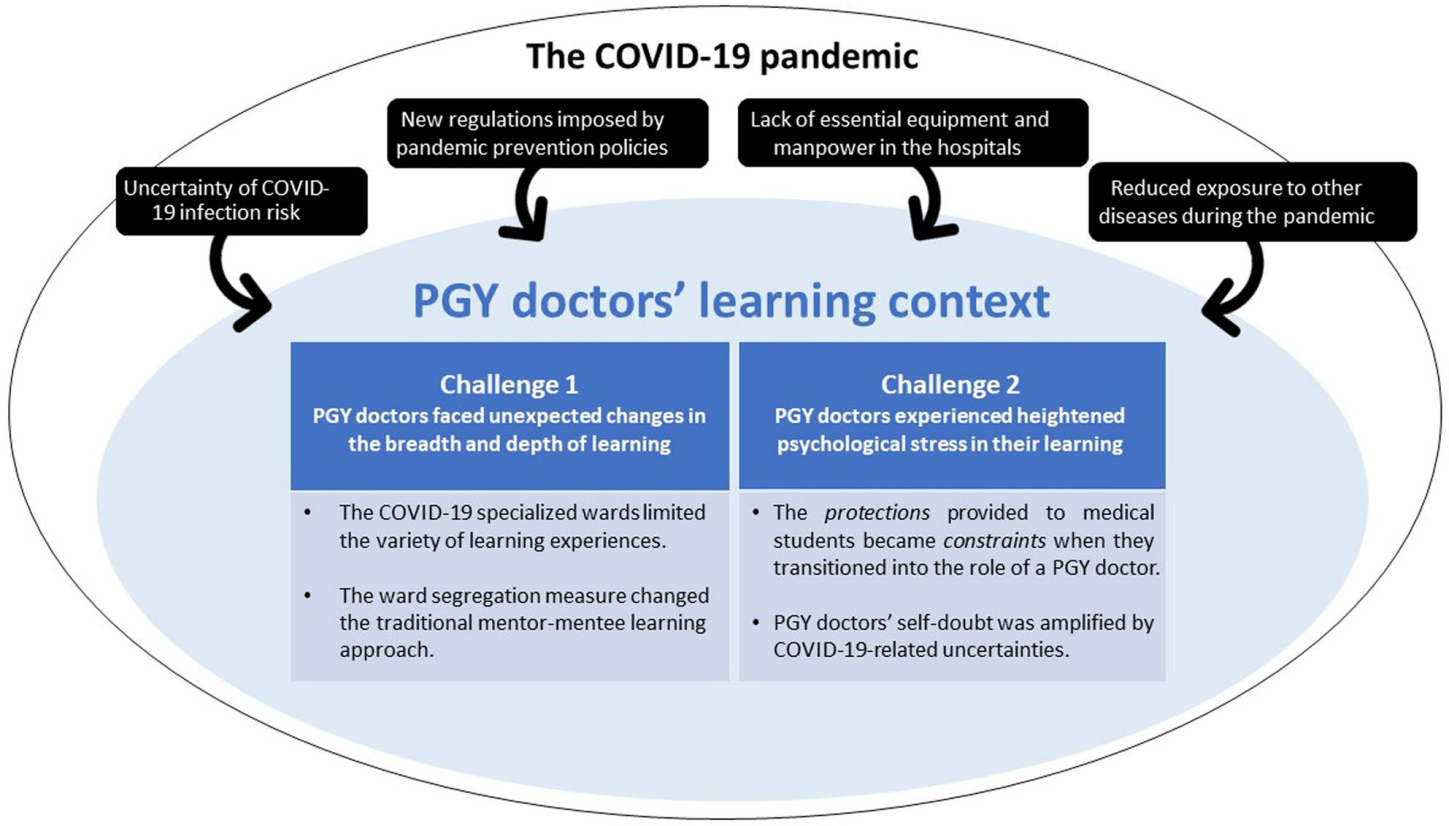
Impacts of the COVID-19 pandemic on PGY doctors’ learning experiences

During the COVID-19 pandemic, hospitals in Taiwan (i.e., the learning context for PGY doctors) underwent considerable changes compared to the pre-COVID era. Through data analysis, four key impacts of the COVID-19 pandemic on the overall learning context were identified, along with two major learning challenges perceived by PGY doctors as a result of these changes (Fig. 2).

### Key impacts of the COVID-19 pandemic on the learning context

#### Uncertainty of COVID-19 infection risk

During the early stages of the pandemic, the general public had a limited understanding of COVID-19 and no vaccines. PGY doctors faced the uncertainty of COVID-19 infection risk. The fear of *“I could be the next one who gets infected”* (Y1-10) was an underlying concern for most participants who still had to come to the hospital to work every day.

#### Required efforts in response to new COVID-19 prevention regulations

Multiple new COVID-19 prevention regulations were implemented due to the Taiwan government’s national pandemic prevention policies. One key policy the government implemented was the mandatory 14-day home quarantine for confirmed cases and their contacts to prevent the spread of the virus. Some participants who had to be quarantined worried about not having sufficient training hours. In terms of the hospital settings, many newly added regulations were often very time-consuming, significantly intensifying the workload. For example, all clinicians were mandated to change protective suits every time when taking care of different patients, and the PGY doctors were responsible for performing pre-admission screenings for all new patients and conducting screening for all the patients when there were new confirmed cases in the same ward. These prevention measures were critical to effectively prevent the spread of the virus in the hospital. However, these new regulations required considerable time and effort from the PGY doctors. In addition, because the pandemic was quickly evolving, government policies and related regulations changed quickly, and as one participant said, *“I was struggling to catch up and adapt to new regulations in a timely fashion”* (Y1-1).

#### Lack of essential equipment and human resource in the hospitals

During the pandemic, many hospitals in Taiwan had to temporarily convert their regular wards with four or six beds into single-occupancy rooms for infection control. However, the equipment, such as computers used to issue medical orders, was often insufficient for the new physical settings. Regarding human resource, before the COVID-19 pandemic, clinicians in Taiwan were mostly responsible for medical tasks, while patients’ family members or hired caregivers handled other activities of daily living. However, due to prevention measures, some participants shared that they were taking care of both types of tasks, which further increased their workload, and as one participant said, *“It was hard to manage so many things at the same time. When the family members were not in the wards*,* it was hard to respond to patients’ needs as prompt as it used to be”* (Y1-17).

#### Reduced exposure to other types of diseases during the pandemic

The reduced exposure to other diseases observed and treated during the pandemic changed the *“learning materials”* for PGY doctors. Due to the fear of contagion, most individuals in Taiwan tended to avoid seeking medical services unless they had urgent health problems (e.g., acute abdominal pain and traumatic injuries). Many scheduled surgeries were also canceled if not urgent. Additionally, due to the comprehensive implementation of infection control measures in the community (e.g., mask-wearing, spatial distancing, and home quarantines), the prevalence of specific transmissible diseases also decreased. For example, the prevalence of enterovirus infections among pediatric cases was typically high during the summer in Taiwan, which was an important part of the training experience. However, during the pandemic, participants reported only a few cases, none of which were severe. Although PGY doctors had more opportunities during the pandemic to learn how to manage new infectious diseases, due to the reduced diversity of diseases, they had fewer opportunities to observe and learn from a greater variety of cases than in the pre-COVID-19 era.

The COVID-19 pandemic had four key impacts on the learning context of PGY doctors. Below, two major challenges that illustrate how these contextual changes caused by the COVID-19 pandemic shaped the learning experiences of PGY doctors are presented 

### PGY doctors faced unexpected changes in the breadth and depth of learning during the pandemic

The breadth and depth of learning appeared to have been significantly impacted by the COVID-19 pandemic in part due to changes in the physical environment. To minimize the risk of virus transmission within hospitals, the Taiwanese government instructed hospitals to (a) establish COVID-19 specialized wards, and (b) implement ‘ward segregation’ measures. These prevention regulations led to unexpected changes to PGY doctors’ learning experiences.

The COVID-19 specialized wards were designed to provide services to individuals who tested positive for COVID-19 and had to undergo a 14-day quarantine. Working in these wards became the primary responsibility of most PGY doctors during the COVID-19 pandemic, reducing their time in other wards for learning. For example, one participant shared that his learning time in other departments had to be shortened: *“For the self-selected training*,* it was supposed to be one month*,* but I only got two weeks. I would say I wasn’t able to learn much because the time was limited (Y2-4)”.* In addition, within these specialized wards, the patients’ symptoms were usually very similar and relatively mild. PGY doctors spent considerable time on the basic management of COVID-19 patients, resulting in a limited breadth of learning experience. As one participant said:

*Most of the cases [in the specialized ward] had similar symptoms. I felt that only one in ten patients had symptoms other than mild respiratory symptoms*,* such as abdominal pain. In terms of my learning*,* the things I could learn were very limited. (Y2-5)*

Another key change to the physical environment as an infection control measure was ‘ward segregation’. This involved dividing wards into several zones and preventing staff cross-movement between zones, except for attending physicians, because their patients might be in multiple zones. Such changes in the physical environment led to changes in learning approaches. Traditionally, the learning approach for PGY doctors mainly relied on close interactions between mentors (i.e., established doctors) and mentees (i.e., PGY doctors). During the two-year training, PGY doctors would rotate through different departments, and in each department, they typically learned from several key mentors. For example, PGY doctors may have shadowed mentors for one to three months, checking on patients in different wards and observing closely how their mentors treated each patient. However, during the pandemic, the ‘ward segregation’ measure significantly changed this traditional learning approach. PGY doctors were required to stay in specific zones to minimize the risk of virus transmission. Therefore, they could not follow their mentors, usually attending physicians, to different zones. Instead, the PGY doctors would interact with multiple attending physicians when they checked on their patients in the assigned zones. However, compared to the traditional mentor-mentee learning approach, the amount of time the PGY doctors interacted with different attending physicians was considerably shorter, and the depth of learning was somewhat reduced. As one participant said:

*Each patient whom I was responsible for was under the care of different attending physicians. Every day*,* we had to report patients’ updates to different attending physicians. In addition*,* during the pandemic*,* patients could come and go from the ward quickly as soon as their quarantine was over*,* and we had to take in new patients. The time we had with each attending physician was quite short. (Y2-3)*

In addition to changes within hospital settings and adjustments to the learning approach, some other learning opportunities typically arranged for PGY doctors were also canceled. For example, some participants mentioned that before the pandemic, PGY doctors were supposed to receive training at different clinical settings. However, these learning opportunities were canceled due to pandemic restrictions and were difficult to reschedule. These unexpected arrangements may limit the breadth of PGY doctors’ medical education and have had long-term impacts on their career paths. As one participant said:

*I was supposed to go on an externship in my sixth year [as a UGY student]*,* but it was canceled because of the first outbreak. Then*,* as a PGY doctor*,* I was supposed to go to the local community to receive different training there. However*,* this opportunity got canceled again due to another outbreak. Now my whole experience has just been limited to the university hospital setting. I’ve never really seen how other hospitals or other settings operate. (Y2-3)*

### PGY doctors experienced heightened psychological stress in their learning due to the pandemic

The psychological stress PGY doctors faced was often related to self-doubt, which arose from two aspects. First, like most PGY doctors in the pre-COVID-19 era, the PGY doctors receiving training during the pandemic also experienced the transition from medical students to newly licensed doctors. As one participant said:

*As a student*,* your main focus was learning*,* and you don’t have many responsibilities…When you become a PGY doctor*,* you are part of the team*,* and you are responsible for the tasks assigned to you. I feel there’s quite a big difference. (Y1-13)*

The transition process itself can be a source of psychological stress, which may have been further amplified by the COVID-19 pandemic. During the UGY stage, many clinical learning opportunities were canceled for protective reasons, resulting in reduced preparatory experiences and limiting the accumulation of clinical experience. Although most participants acknowledged that these cancellations were unavoidable, they nonetheless felt less prepared for the demands of subsequent PGY training. As one participant whose clinical training was canceled due to the pandemic said:

*The physician taught us infectious diseases using lectures in classrooms instead [of direct observations in the clinical settings] … However*,* I feel there’s still a gap between having lectures in the classroom and having direct interactions with actual patients. (Y1-4).*

Another participant shared that when she was a UGY student, she had no opportunity to directly observe how clinicians cared for patients with COVID-19 because UGY students were not permitted to enter wards with confirmed cases. However, once she became a PGY doctor, she was immediately expected to work on the frontline:

*There is a big difference between being a clerk and a PGY doctor…As a clerk*,* you’re a student*,* and they [the staff] always prioritize protecting the students. If you’re a PGY doctor*,* you must go directly to the front line. No-one would tell you that you can stay at home and not work [laughs] (Y2-5).*

In other words, the *protections* provided to them as UGY students became *constraints* when they transitioned into the role of a PGY doctor. This gap further amplified the self-doubt and psychological stress during the transition from medical students to newly licensed doctors, as illustrated by one participant’s perceived mismatch between expected and actual capabilities while working in the emergency department (ED):

*Typically for clerks*,* they should be able to go to the ED and learn*,* but I did not have any prior experience in the ED [due to the pandemic]. However*,* as a PGY doctor*,* I was expected to take on clinical work immediately…There were many procedures that I hadn’t practiced as a clerk before. I had to first spend several days exploring so I wouldn’t act “clumsy” in the ED…I think there was definitely a gap. (Y1-15)*

Second, as frontline workers during the pandemic, PGY doctors often faced unprecedented challenges that those in the pre-COVID era had not encountered. The COVID-19 pandemic increased uncertainty in performing clinical tasks, which further intensified PGY doctors’ pre-existing self-doubt. Some participants reported feeling less confident performing routine examinations while wearing additional protective equipment. For instance, during an electrocardiogram (ECG) examination in the specialized ward, the heavy protective suits reduced tactile sensitivity and made it difficult to apply ECG electrodes, and as one participant said: *“I think following these procedures under COVID-19 is challenging. Wearing gloves and putting on protective suits…it’s really tough to perform these [examination] tasks*,* and it could lead to significant issues”* (Y1-12). In addition to the unfamiliarity with routine examinations involving protective equipment, the continuously changing prevention policies also posed unprecedented challenges in clinical settings. Such situations may have further intensified the psychological stress stemming from self-doubt among PGY doctors. For example, Y1-1 recalled a time when he encountered an emergency in the specialized ward where a family member of one patient fainted suddenly, and he had to deal with the situation on his own:

*After all*,* the PGY doctors have just started. There are doubts about your diagnosis and judgment skills…and really big doubts about yourself. You’re not really familiar with making diagnoses…and even if everything looks the same as what’s being said in the textbooks*,* you don’t dare to make decisions with 100% certainty. I felt the case might be handled much more smoothly if I could get immediate support [while dealing with the family member who had fainted]. (Y1-1)*

## Discussion

Five focus groups were conducted with 24 PGY doctors in Taiwan to explore their learning experiences during the COVID-19 pandemic. Regarding their learning context (i.e., the hospital environment), four key impacts of the COVID-19 pandemic were identified, including (a) facing the uncertainty of COVID-19 infection risk, (b) new regulations imposed by pandemic prevention policies, (c) lack of essential equipment and personnel in hospitals, and (d) reduced exposure to other diseases observed and treated during the pandemic. The pandemic’s changes influenced the PGY doctors’ learning process and led to two main challenges. First, they faced unexpected changes in the breadth and depth of learning. Second, they experienced heightened psychological stress in their learning.

Consistent with previous studies, the COVID-19 pandemic altered hospital environments, with healthcare systems in many countries undergoing rapid operational changes, including physical reorganization of workspaces and adjustments to staffing models [32, 33]. Prior research has commonly highlighted shortages of medical staff and personal protective equipment (PPE) as major resource constraints [34–38]. In contrast, PGY doctors in the present study emphasized a lack of caregiver support due to infection prevention regulations, which increased their workload, rather than shortages of medical staff. Participants also reported no significant concerns about PPE availability, likely reflecting Taiwan’s relatively mild pandemic conditions and improved preparedness following the 2003 SARS outbreak. Instead, a key challenge was unfamiliarity with performing clinical tasks while wearing PPE, which heightened anxiety and reduced confidence. These findings indicate that medical educators should integrate PPE-related clinical practice into PGY training even in the absence of acute public health emergencies. Prior research suggests that proactively preparing learners for potential challenges enhances their ability to respond effectively in high pressure clinical situations [13, 39, 40].

During the pandemic, participants in the present study felt that both the breadth and depth of their learning were constrained, including reduced clinical exposure, cancellation of learning opportunities, and reduced access to mentor-mentee interactions. Similar concerns have been reported in prior studies. For example, Coleman et al. [41] reported that surgical trainees worried about their reduction in operation volume and inability in fulfilling minimum case requirements. A scoping review of postgraduate medical education in the UK also highlighted negative impacts on PGY doctors’ career development and increased feelings of isolation from senior clinicians during the pandemic [34]. In response, health authorities and medical educators may need to reconsider the education continuum for PGY doctors to increase clinical exposure and expand access to diverse learning models. Proposed strategies include greater use of simulation-based education, re-establishing ‘firm-based’ team structures to compensate for missed rotations, integrating online learning, and facilitating learning through telephone clinics [42, 43].

Previous studies have consistently shown that health professionals’ psychological well-being deteriorated under pandemic-related pressures [41, 44, 45]. Building on this literature, the present study further elaborated on how the COVID-19 pandemic cumulatively intensified psychological stress among PGY doctors. This stress mainly arose from insufficient clinical experience because many learning opportunities were reduced for protective reasons during undergraduate education. When PGY doctors later faced COVID-19-related uncertainty, their awareness of these experiential gaps amplified self-doubt, turning earlier protections into constraints. By clarifying the potential mechanisms underlying heightened psychological stress, these findings highlight opportunities for medical educators to address core issues. Rather than focusing solely on protecting undergraduate medical students, it may be more practical to provide structured guidance that enables meaningful clinical training while ensuring safety. For example, the UK prioritized clinical training for final-year and senior medical students, emphasizing student safety, adequate PPE, and adherence to distancing guidelines [46]. Similarly, the United States facilitated clinical training in low-risk communities and ensured sufficient PPE when exposure was unavoidable [46]. These approaches align with prior evidence that medical students were willing to train during the pandemic and could benefit from such experiences [11], which also serve as an essential foundation for subsequent PGY training.

The results showed that PGY doctors experienced psychological stress as they faced both self-doubt and pervasive uncertainty, requiring considerable mental resilience to keep learning. Future postgraduate medical education needs to better prepare PGY doctors to manage stress and make clinical decisions under uncertainty during public health emergencies. Mental resilience programs may help strengthen PGY doctors’ well-being; for example, stress management and resiliency training [47, 48] and the ‘Resilience on the Run’ program [49] have been shown to improve mental health among medical interns. At the institutional level, clear clinical practice guidelines may also reduce stress related to clinical decision-making [7].

### Limitations and future research

There are three major limitations in the present study. First, all interviewed PGY doctors were from the same medical center in Southern Taiwan. Therefore, the results may not apply to PGY doctors who received clinical training in other hospitals. Accordingly, future studies may want to explore the experiences of PGY doctors who received training in other types of hospitals from different regions. Second, the research team did not discuss the interpretation of the data with the participants to clarify if the findings accurately reflected their experience. To better understand the impact of future public health emergencies on medical training, a targeted survey could be developed to help medical educators and policymakers respond in real time. Such a survey should assess disruptions to the learning context, including safety concerns, new regulations, resource limitations, and changes in case variety. In addition, given the findings highlighted that impacts differ by training stage and that learning experience during the UGY stage may influence subsequent PGY training, the survey should include separate sections for UGY students and PGY doctors. For UGY students, the survey should examine which clinical observation opportunities have been canceled and identify critical experiences that need to be supplemented in future training. For PGY doctors, the survey should explore temporary shifts in clinical responsibilities and learning methods, such as disruptions to traditional mentor-based learning models, and document any training opportunities lost due to emergency-related role changes. Such timely information would enable comparison between PGY doctors affected by public health emergencies and those who were not, facilitating targeted compensation for missed training. In terms of psychological impact, attention should be given to levels of self-doubt and perceived isolation experienced during such crises. Third, the COVID-19 infection control policies and infrastructures supporting virtual medical education were different across countries. Considering the different medical training systems worldwide, some findings from the present study might not generalize to other countries. Therefore, research based on cross-country data is needed to compare the differences across countries. Moreover, future studies may consider using innovative and engaging teaching–learning methods to facilitate medical education learning [50].

## Conclusion

The COVID-19 pandemic greatly affected doctors receiving postgraduate medical education. The results of five focus groups comprising PGY doctors exploring their learning experiences suggested that the pandemic posed unavoidable changes to the learning context and new learning challenges for PGY doctors These highlighted that the *protections* they were given as medical students became *constraints* while transitioning to PGY doctors, and that the reduced training opportunities in different clinical settings, as well as the limited experience in the traditional mentor-mentee learning approach, may lead to changes in the breadth and depth of learning. The results suggest the need for medical educators to create and provide alternative learning opportunities to enhance the capabilities and confidence of PGY doctors during future epidemics and pandemics.

## Supplementary Information

Supplementary Material 1.

## Data Availability

The datasets generated and/or analyzed during the current study are not publicly available due to concerns of privacy and confidentiality but are available from the corresponding author upon reasonable request.

## Abbreviations

COVID-19: Coronavirus disease 2019
UGY: Undergraduate year
PGY: Postgraduate year
PPE: Personal protective equipment
