# Supplementary material for: Protection or constraint? A phenomenological study of the learning experience among doctors receiving postgraduate medical education during the COVID-19 pandemic
Source: BMC Med Educ. 2026 Feb 12;26:438. doi: 10.1186/s12909-026-08782-y (PMC12998013; doi:10.1186/s12909-026-08782-y)
Supplement: Supplementary file 1 — Supplementary Material 1. [file 12909_2026_8782_MOESM1_ESM.docx]

Supplementary material: Focus group interview guide

1. In terms of your clinical work, how do you feel about the COVID-19 pandemic?
   - How do your family members or friends feel about it?
2. What COVID-19-related tasks are you responsible for in your clinical work?
   - How is the increased risk due to the COVID-19 pandemic managed in clinical settings?
   - What kinds of actions do you take in response to the increased risk?
   - What are the significant challenges you encountered or are encountering during the COVID-19 pandemic?
   - How do you cope with it?
   - Is there any factor that makes it more difficult or easier for you while dealing with the challenges?
3. How do you find your clinical training impacted by the COVID-19 pandemic?
   - What are the significant challenges you encountered or are encountering?
   - How do you cope with it?
   - Is there any factor that makes it more difficult or easier for you while dealing with the challenges?
